# Optimal CRT Implantation—Where and How To Place the Left-Ventricular Lead?

**DOI:** 10.1007/s11897-021-00528-9

**Published:** 2021-09-08

**Authors:** Christian Butter, Christian Georgi, Martin Stockburger

**Affiliations:** 1 Department of Cardiology, Heart Center Brandenburg, University Hospital Brandenburg, Brandenburg Medical School Theodor Fontane, Brandenburg, Germany; 2 Faculty of Health Sciences Brandenburg, Brandenburg Medical School Theodor Fontane, Brandenburg, Germany; 3 Department of Internal Medicine/Cardiology, Havelland Kliniken GmbH, Nauen, Germany

**Keywords:** Cardiac resynchronization therapy, Lead implantation, Acute hemodynamic response, Multimodal imaging, Coronary sinus

## Abstract

**Purpose of Review:**

Cardiac resynchronization therapy (CRT) represents a well-established and effective non-pharmaceutical heart failure (HF) treatment in selected patients. Still, a significant number of patients remain CRT non-responders. An optimal placement of the left ventricular (LV) lead appears crucial for the intended hemodynamic and hence clinical improvement. A well-localized target area and tools that help to achieve successful lead implantation seem to be of utmost importance to reach an optimal CRT effect.

**Recent Findings:**

Recent studies suggest previous multimodal imaging (CT/cMRI/ECG torso) to guide intraprocedural LV lead placement. Relevant benefit compared to empirical lead optimization is still a matter of debate. Technical improvements in leads and algorithms (e.g., multipoint pacing (MPP), adaptive algorithms) promise higher procedural success. Recently emerging alternatives for ventricular synchronization such as conduction system pacing (CSP), LV endocardial pacing, or leadless pacing challenge classical biventricular pacing.

**Summary:**

This article reviews current strategies for a successful planning, implementation, and validation of the optimal CRT implantation. Pre-implant imaging modalities offer promising assistance for complex cases; empirical lead positioning and intraoperative testing remain the cornerstone in most cases and ensure a successful CRT effect.

## Introduction

Cardiac resynchronization therapy (CRT) might be judged as one of the most impressive success stories for heart failure treatment since its initial reports nearly 30 years ago.

Driven by the concept and patent by Dr. Mower in 1990 and the first encouraging case report from Serge Cazeau in 1994 using a four-chamber stimulation with two Y-adapters and an epicardial left ventricular lead, the understanding of conductance disorder, especially left bundle branch block (LBBB), grew immediately [1, 2]. Furthermore, technical tools were developed to access the coronary sinus, to reach challenging targets in the tributaries, and to ensure stable lead position and allow device programming, which is adapted to the individual electro-mechanical disorder. Based on our current knowledge, this first patient with a QRS of 200 ms, LBBB, non-ischemic cardiomyopathy, and severe heart failure is reflecting a typical “responder.”

With the extension of CRT therapy and growth of scientific knowledge, the question came up what the definition of “response” is and when and how it is determined. For years, the statement that nearly 30% of CRT patients do not respond to resynchronization therapy has become general knowledge and is discussed and partially accepted without questioning the reasons. Generally, the main goals of HF treatment are relief of symptoms, restoration of quality of life (QOL), slowing of disease progression, lowering the rates of emergency room visits and hospitalizations, and prolongation of life. Alleviation of symptoms and lower morbidity are the main objectives for patients in New York Heart Association (NYHA) HF functional Classes III and IV and preventing disease and HF progression is priority for patients in NYHA Classes I and II [3].

The further the endpoint of judgment veers away from the time of CRT implantation, the more it will be overlapped by other events, disease progression, or other concomitant factors that overlap the CRT effect. The time after implantation, which best reflects the CRT effect, has never been defined and will clearly influence the interpretation of trial data.

Furthermore, patients’ expectations in CRT therapy regarding response will differ from scientific judgment. Whereas the patient expects an improvement in quality of life, in physical activity, and decrease in hospitalizations, he will not necessarily be convinced by the improvement of echocardiographic remodeling measures or not even by prolongation of his life in poor quality. Without regard to the discussion of response or non-response, the basis for a potential improvement is always the implantation of the device and the definition of the “optimal left ventricular pacing site.”

## Finding the Optimal LV Lead Position

Up to now, achieving synchrony between both ventricles as well as normalizing right and left ventricular filling is mainly facilitated by the implantation of a right atrial, right ventricular, and left ventricular transvenous epicardial lead using the coronary system.

An unchangeable limitation in selecting the optimal implantation site is the given anatomy of the coronary venous system, which varies substantially. Whereas in some patients different site branches might be technically accessible, the implanter might be glad in others if only one tributary will allow a stable lead position close to the intended target area however this is defined. Finally, the LV lead position is often a compromise between target area, lead stability, threshold, intrinsic signal, and inadvertent diaphragm stimulation. Especially in patients who already had cardiac surgery, aortal coronary venous grafts, or coronary venous lead extractions, the choice is limited. There are multiple tools and tricks to overcome these problems [4].

The process of defining the optimal site for left ventricular lead placement might be divided into different steps:

1. Understanding the individual problem of conduction delay (LBBB) including underlying heart disease, medical history, and previous interventions.
2. Characterizing the quality of tissue, including the detection and visualization of areas of scar, late(st) electrical and mechanical activation—PRIOR Implantation NON-INVASIVELY.
3. Showing the implanter’s reality by coronary sinus venogram and estimating the accessibility of target tributaries based on experience and available tools, pure anatomical approach—DURING implant.
4. Detecting and correcting electrical and mechanical left ventricular delay including acute hemodynamic optimization—DURING implantation INVASIVELY.

**Ad 1:** **Understanding the individual problem of conduction delay (LBBB) including underlying heart disease, medical history, and previous interventions.**

When considering a patient for CRT, a global understanding of the underlying mechanisms of heart failure is needed and all reversible and treatable targets as ischemia, valvular disease, metabolic disorder, arrhythmias including atrial fibrillation etc. should be eliminated first. Guideline-directed heart failure medication should be optimized for at least 3–6 months, including modern strategies as ARNI and SGLT2 inhibitors before re-evaluating the need for CRT using echocardiography and the clinical estimation [5]. As far as the patient suffers from a non-ischemic cardiomyopathy, shows a QRS complex > 150 ms with typical LBBB morphology, and has not undergone any previous cardiac surgery, the coronary venous system usually offers at least a few options of tributaries. The LV lead implant site will be less crucial in this population and a good response can be predicted.

In contrast, patients with an ischemic origin of their heart failure, multiple coronary interventions, previous coronary artery bypass grafting (CABG), QRS complex between 130 and 150 ms, or non-LBBB configuration likely have a more challenging coronary sinus anatomy with less options to optimize the LV lead position. A response in these patients is potentially difficult to predict and more preceding imaging methods such as MRI, strain analysis, and electroanatomical mapping should be integrated to identify the best pacing site. Furthermore, additional information regarding the acute response (see 4.) will be helpful to decide whether an alternative approach should be preferred.

The same strategy is recommended for CRT reimplantation or chronic non-responder.

**Ad 2**:**Characterizing the quality of tissue, including the detection and visualization of areas of scar, late(st) electrical and mechanical activation—PRIOR Implantation NON-INVASIVELY.**

Preceding non-invasive imaging is helpful to characterize the tissue quality of the myocardium, detect scar and viable myocardium, and detect regions of delayed electrical and mechanical activation. The detection of intramural scar and its distribution within and over the left ventricular wall can add additional information about the risk of sudden cardiac death in dilative cardiomyopathy and support the decision to implant a CRT-D instead of CRT-P.

### Cardiac MRI (CMR) and Dual-Source CT (DSCT)

Late gadolinium enhancement (LGE) CMR is the gold standard for delineating myocardial scar with high resolution, as the superior spatial resolution of LGE-CMR permits differentiation between epicardial, transmural, and sub-endocardial infarction.

The extent, location, and transmurability of scar is of major importance for the guidance of the implantation site and the outcome independent of its ischemic or non-ischemic origin [6–8]. The size of scar is inversely correlated with the acute response, reverse remodeling, functional improvement, and survival. A lead implantation in the empirically targeted postero-lateral region will lead to a lower response rate and a potential increase of arrhythmias if it expands the scar [6]. Both CMR-guided last mechanical activation as well as electrical approaches have been focusing on detection of the latest left ventricular activation and considering this an optimal pacing site [9, 10]. If this is found within a larger transmural scar region, it seems questionable to be the preferable site. Sometimes electrical activation propagating from off the most delayed spot might result in an earlier mechanical activation restoring synchrony when thus avoiding a scar region [11]. This correlation between scar morphology and subendocardial, subepicardial, or transmural dispersion and mechanical response will at least help to avoid certain regions and target viable border zones. Approximately one third of CRT candidates are not eligible for an MRI due to metallic implants or other rhythmological devices, and other imaging technologies have to be discussed. Modern dual-source CT (DSCT) scans can detect scar, but its information regarding tissue characterization and viability is less compared to MRI. Besides, DSCT is not delivering superior information about delayed mechanical activation and carries the risk of contrast and radiation exposure.

Summarizing, CT is infrequently used outside from scientific questions to achieve an anatomical overview of the vascular access and the coronary venous anatomy [12, 13], especially in patients with congenital heart disease or previous cardiac surgery. Its role in multimodality imaging in planning and performing CRT implantations will be discussed later.

### Trans-Thoracic Echocardiography

Transthoracic echocardiography plays a major role in imaging of heart failure patients, not only scheduled for CRT. Due to its cost-effectiveness, its general availability, and its fast information, it should be used in every CRT candidate. Scar is detected by wall thinning and abnormal motion patterns, and viable and ischemic myocardium can be identified as well.

New modalities such as 3D contrast echo and pulse cancelation have been investigated to improve scar detection [14, 15].

Furthermore, concomitant problems associated with asynchrony like mitral regurgitation can be judged immediately. Since the early days of CRT, multiple echocardiographic parameters and indices have been investigated to qualify and quantify disturbed and delayed wall motion specially to localize the area of latest mechanical activation.

Early investigations started with tissue Doppler imaging (TDI), established a dyssynchrony index (SDI), and continued detection of asynchrony with speckle tracking technology using radial and longitudinal strain. These trials were retrospective and/or single-center investigations and showed that these parameters can either identify patients for CRT or predict response. Also, prospective multicenter trials which used these parameters to identify asynchrony according to these definitions failed to show the benefit of the echocardiographic guided implantation, though. Especially in patients with a QRS < 130 ms, a CRT treatment deteriorates outcome, despite the evidence of echocardiographic proven dyssynchrony [16, 17].

There are currently no data showing that *intraprocedural* echocardiography either transthoracically or transesophageally applied used during the CRT implantation and during the LV lead placement is useful to select the best tributary to reduce asynchrony.

*Preprocedural* identification of delayed contraction by radial strain and targeting this region for the final LV lead position resulted in TARGET and STARTER in a reduction of asynchrony, reverse remodeling, and diverging results regarding all-cause mortality and CRT-D free survival compared to an empiric LV lead placement [18, 19]. However, it has to be emphasized that in none of these randomized trials the LV lead position was investigated in different positions and intra-individually adapted or optimized according to intraprocedural echo parameters. Currently, no echo parameter is accepted or guideline endorsed for the identification of CRT responders [20]. This is also reflected in the 2016 ESC guidelines for the diagnosis and treatment of acute and chronic heart failure, which do not recommend using the presence of echocardiographic dyssynchrony as selection criteria for CRT [21].

### Electrocardiography

Twelve-lead ECG is the traditionally and currently mainly used non-invasive method to diagnose electrical disorders and conduction disturbances. It is the easiest tool to detect left bundle branch block (LBBB) and select patients for CRT. The expansion up to 300 electrodes allows a 3D visualization of onset of activation and propagation captured from the body surface [22]. A correlation of low voltage and scar has been shown [23, 24]. Furthermore, the change of electrical activation by CRT can be detected, but no data are published showing that the spatial and temporal resolution of the electrocardiographic imaging (ECGi) is high enough to be used as guidance for the best LV lead spot during the implantation.

### Multi-Modality Imaging and Image Fusion Technology

**Fig. 1:**
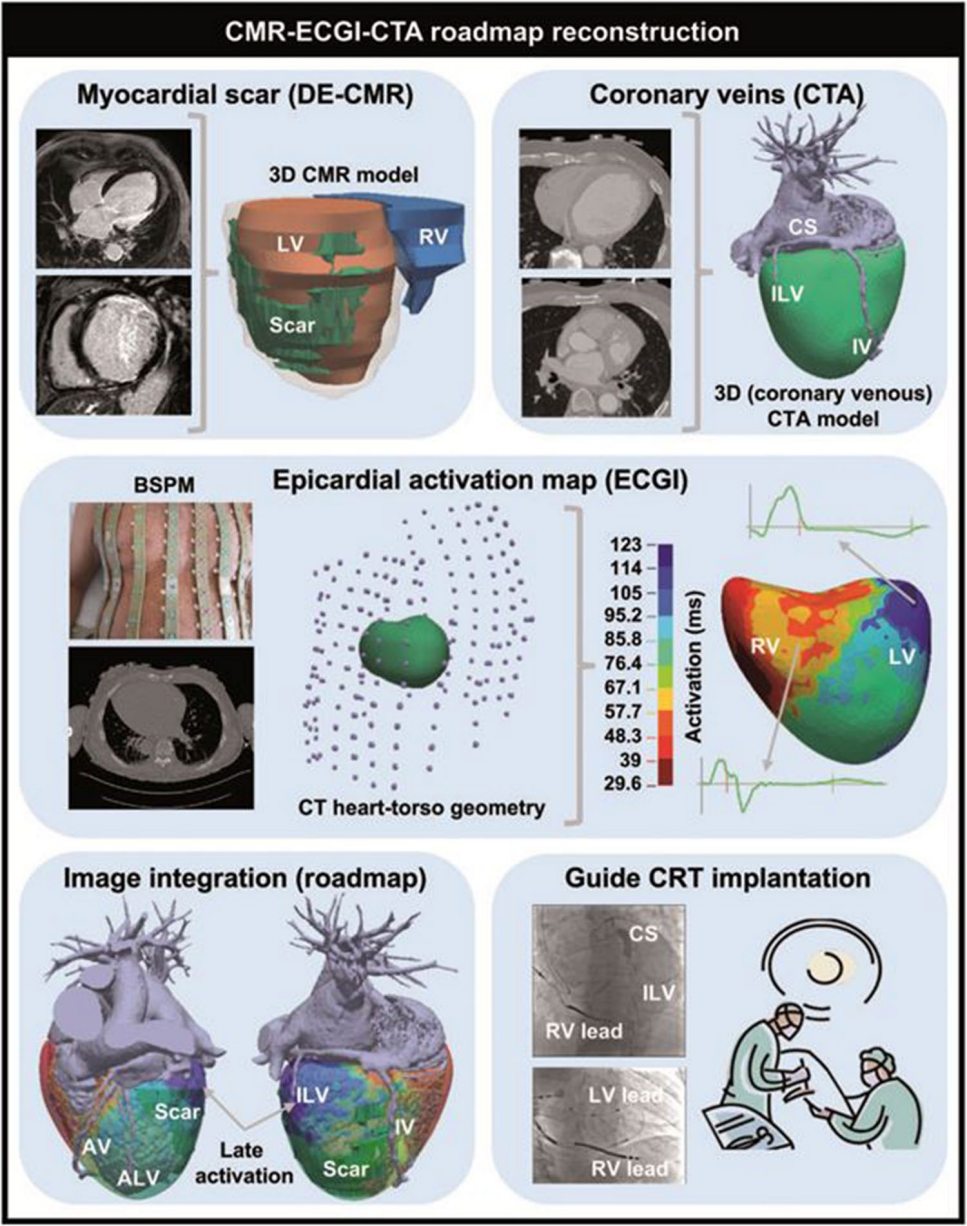
CMR–ECGi–CTA roadmap reconstruction [25]. Workflow for CMR-ECGI-CTA roadmap reconstruction for CRT implantation guidance. AV, anterior vein; BSPM, body surface potential measurement; CMR, cardiac magnetic resonance imaging; CS, coronary sinus; CTA, computed tomography angiography; DE-CMR, delayed enhancement cardiac magnetic resonance imaging; ECGI, electrocardiographic imaging; ILV, inferolateral vein; IV, inferior vein; LV, left ventricle; RV, right ventricle

The so-called multi-modality imaging approach is based on the fusion of different imaging modalities to match information about tissue characterization (MRI; LGE) especially scar and viability (PET-CT), late mechanical or electrical activation (MRI, Echo; speckle tracking, ECGi), and its correlation to the given coronary venous anatomy (CT; MRI). This information will be fused with the CS angiography to guide the implanter to select the optimal side branch for LV lead [9, 25, 26•] (Fig. 1).

CT is superior in delineating the coronary venous tree with a higher spatial resolution compared to MRI, especially if rapid acquisition is performed in 3D technology in whole heart cycles.

This approach optimizes the efforts of non-invasive imaging, although it is expensive and time consuming and is hardly covered by the reimbursements for CRT implantations in most countries of the world—apart from clinical trials.

Disappointingly, MRI or radial strain-guided LV lead placement, in combination with multimodality imaging, did not result in increased clinical or echocardiographic response, nor in a significant reduction of death or heart failure hospitalization. However, only 77% of all patients had an optimal or adjacent LV lead placement. Thus, cardiac CT can be valuable for identification of suitable cardiac veins at the free LV wall, but reaching the intended target branch with a stable fixation remains the limiting factor as previously shown in STARTER (86% concordant) and TARGET (63% concordant) and Borgquist et al. (77%) [18, 19, 27•].

**Fig. 2:**
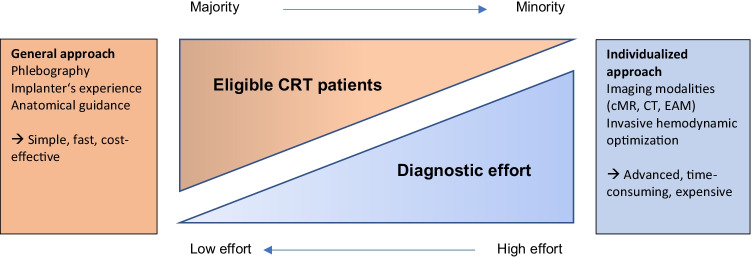
Proportion of CRT patients and reasonable diagnostic effort. The majority of CRT-eligible patients can be treated adopting a general approach. For selected patients and complex cases, an individualized approach using pre-implant imaging or invasive hemodynamic optimization is needed. cMR, cardiac magnetic resonance; EAM, electroanatomical mapping

Summarizing, standard use of a targeted multimodality imaging strategy should currently not be recommended for all CRT patients but may prove valuable in select cases. Skilled implanters, easy-to-use image integration tools, and segmental evaluation strategies are all important for multimodality imaging to reach its full potential to facilitate targeted CRT implantations (Fig. 2).

**Ad 3**:**Showing the implanter’s reality by coronary sinus venogram and estimating the accessibility of target tributaries based on experience and available tools, pure anatomical approach—DURING implant.**

Generally, the implanter of a CRT device should not restrict himself to the pure mechanical adjunct but should always have a general knowledge of the patient’s medical history, the underlying cause of heart failure, and at least basic information derived from transthoracic echo and 12-lead ECG. In most routine cases, the initial intraprocedurally performed coronary sinus venogram gives the first information (or surprise) about the anatomy and uncovers the challenges to target a certain tributary. An occlusive balloon venography delivers a more detailed angiogram and allows an estimation which tributaries might be reached based on the implanter’s skills and his knowledge about tools and tricks to finish successfully [4].

**Fig. 3:**
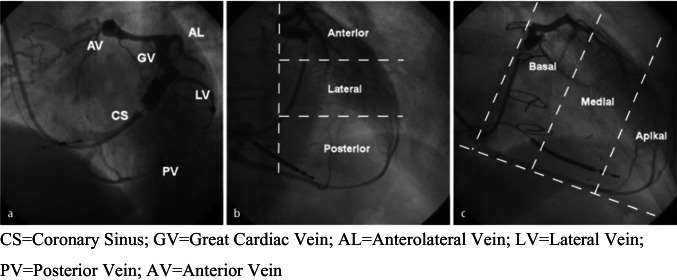
Angiographic classification of the cardiac venous anatomy in LAO (**a**, **b**) and RAO (**c**) projection. CS, coronary sinus; GV, great cardiac vein; AL, anterolateral vein; LV, lateral vein; PV, posterior vein; AV, anterior vein

According to the anatomical classification from the implant venograms used in MADIT-CRT, the final lead tip position was allocated to basal, midventricular, and apical and anterior, lateral, and posterior and correlated with outcome parameters (see Fig. 3). This retrospective analysis clearly demonstrates that an apical LV lead position is associated with an increased heart failure hospitalization and death in all subgroups [28]. A difference between an anterior, posterior, and lateral position could not be detected. Consequently, an apical position should be avoided anyhow by selecting a lead type and a tributary in which it can be fixed in a midventricular position.

In a further data analysis of MADIT-CRT, focusing of LBBB patients’ posterior or lateral LV lead location was associated with significant long-term mortality reduction, accompanied by LV reverse remodeling. The deleterious effect of an apical position was confirmed [29••].

A recently published single-center retrospective analysis of more than 2000 CRT recipients who were followed for nearly 4 years showed a significant long-term mortality benefit for a lateral placement over an anterior or posterior lead position [30].

Derived from these studies, an anatomically guided lateral lead position is never unfavorable, even advantageous in LBBB and should be the primary intended LV lead site, always avoiding being located close to the apex.

This purely anatomically guided implant strategy is mainly used in daily routine when a straightforward quick implantation is intended.

A more individual strategy should be chosen to optimize the LV lead position in complex patients with QRS < 150 ms, scar areas, and a difficult venous anatomy offering different side branches.

**Ad 4:** **Detecting and correcting electrical and mechanical left ventricular delay including acute hemodynamic optimization—DURING implantation INVASIVELY.**

**Fig. 4:**
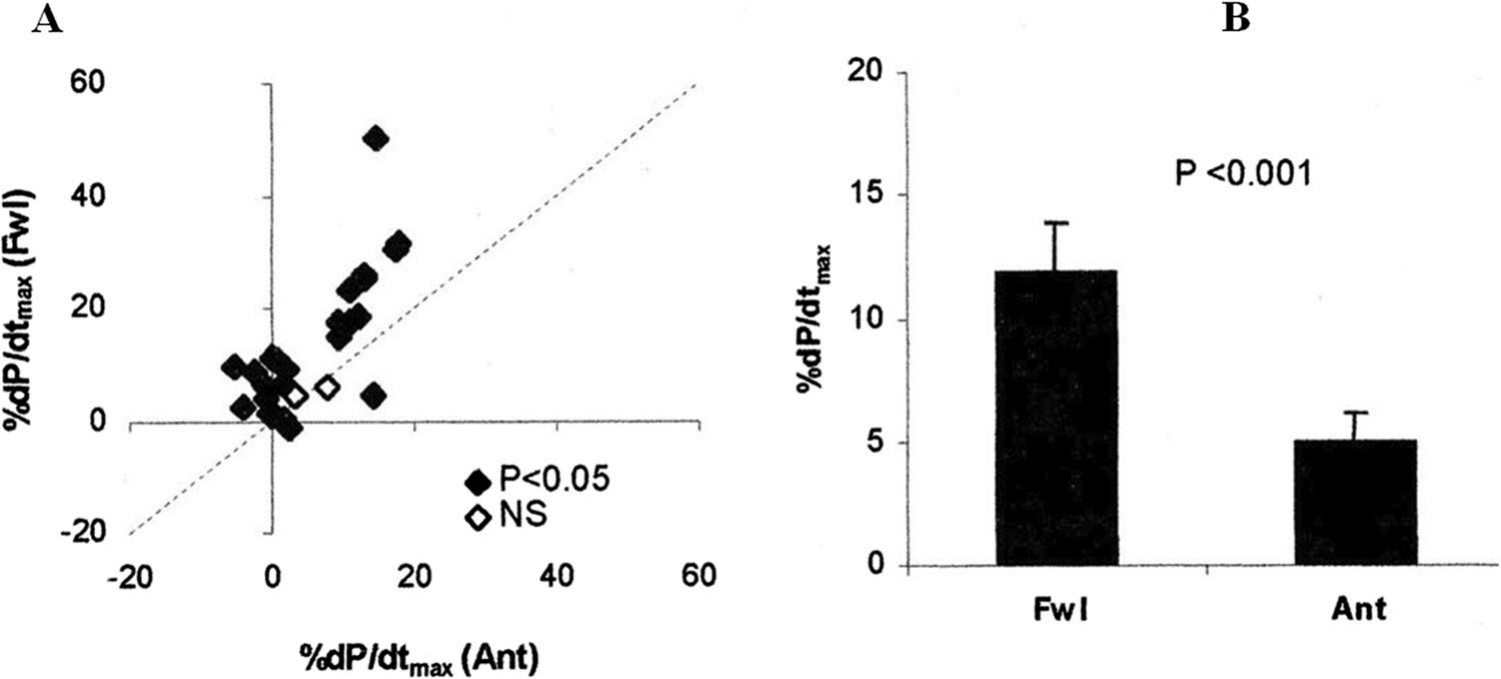
Acute hemodynamic effects of free wall (Fwl) and anterior (Ant) pacing [31]. **A** Scatter plot comparing %dP/dtmax with Fwl and Ant stimulation. Each point (*n* = 30) is the response for 1 patient at the optimum AV delay. Symbols represent individual patients who experienced a significant (♢) or non-significant (♦) difference between Fwl and Ant stimulation response. Points above the identity line (dashed) have a larger Fwl stimulation response. **B** Summary data demonstrating significant LV + dP/dtmax benefit of Fwl versus Ant stimulation for all patients (*n* = 30, *P* < 0.001)

Early in CRT evolution, acute hemodynamic measurements showed that hemodynamic response is highly related to different pacing sites [31–33]. Stimulation of either LV alone or BIV in a VDD mode using the epicardial venous access resulted in a superior effect using the free wall (what is lateral or posterolateral) compared to an anterior (via the intervententricular branch) stimulation site [31] (Fig. 4). Different tools have been used over the years to measure either changes in LV + dp/dt, which reflects the maximum change in LV contractility or the change in pulse pressure (PP), which is a parameter of change in forward stroke volume. A dual transducer micromanometer catheter (SPC-780c; Millar Instruments) can simultaneously measure both parameters; a pressure wire protected by a guiding catheter and placed in the LV (usually used for intracoronary FFR measurement) can be used for dp/dt changes alone. The simplest and cheapest tool is a fluid-filled pigtail catheter in the LV and an arterial sheath to determine either the best pacing site or the best AV-delay. It has to be emphasized that every pacing site is associated with an optimal AV-delay, thus an individualized protocol has to be run. Every patient has an individual superior pacing site.

Whereas the average LV + dp/dt increased at the free wall by 14%, it was 6% at the anterior site. Pulse pressure improved by 8% vs. 4%. LV and BIV pacing effects were similar [31] (Fig. 4).

Notably, one third of the patients showed a deterioration of contractility when paced anterior, but in no patient a negative hemodynamic effect appeared at the free wall.

This clearly demonstrates that it would be worth to identify the individual optimal pacing site and AV-delay.

A hemodynamically optimized chronic LV pacing alone showed in a 3-month cross-over design significant functional improvements in the wide QRS group (> 150 ms). The improvements were comparable in a similar study design for BIV stimulation without preceding invasive testing [1].

A LV + dp/dt response of > 10% during implant predicts a LV reverse remodeling at 6 months and thus will help to guide the lead placement [34]. Nevertheless, the baseline LV + dp/dt seems to be a better predictor for 1-year survival than the acute response using a cut-off of 650 mmHg/s [35]. These findings are not generally conflicting because baseline LV + dp/dt is a measure of global impaired contractility before CRT and is furthermore influenced by other factors like mitral regurgitation which is also substantial for survival [36]. Still, there are no arguments imaginable not to target the site with the best relative improvement.

**Fig. 5:**
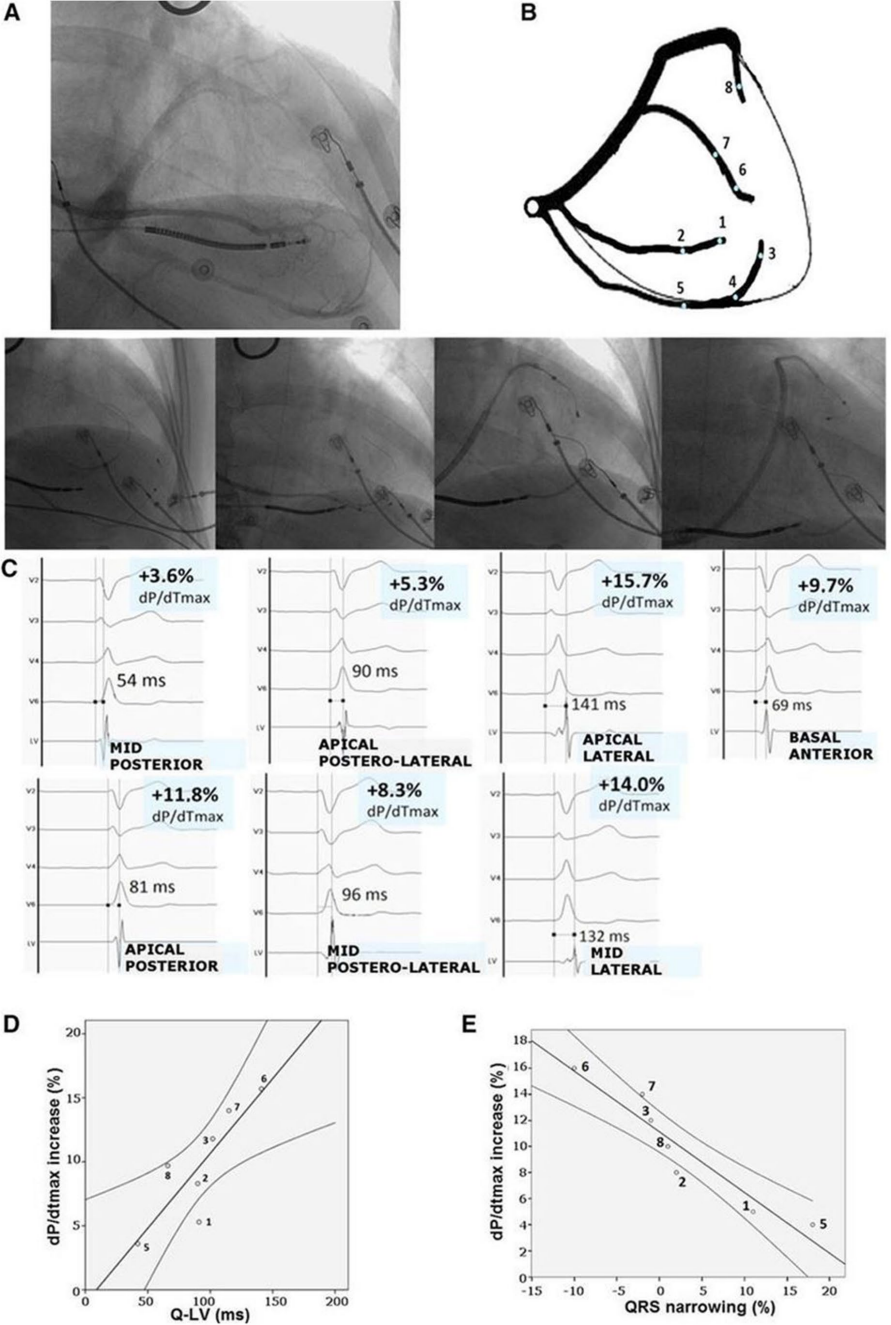
Coronary sinus anatomy and Q-LV intervals at different pacing sites [40]. In a 59-year-old man with non-ischemic cardiomyopathy, New York Heart Association Class III, chronic atrial fibrillation, left ventricular (LV) ejection fraction 20%, left bundle branch block, and QRS 180 ms, 4 veins and 8 pacing sites were tested. **A** Venous angiography. **B** Schematic representation of the venous anatomy and pacing sites. **C** Q-LV measurements and increase in LV dP/dtmax are displayed for 7 available pacing sites (site no. 4 was discarded owing to elevated pacing threshold). **D** Correlation between percentage increase in LV dP/dtmax and Q-LV interval. **E** Correlation between percentage increase in LV dP/dtmax and QRS narrowing

Based on our global understanding for conduction delay, it was predominantly the goal to identify the region of latest electrical activation (LEA) [37, 38]. Whereas electro-anatomical mapping is cost intensive, time consuming, and has not made its way into the routine CRT implantation procedure, detection of LEA is simple, cheap, and can be used intraprocedurally without any other mapping equipment [39]. The delay can be evaluated at different sites according to the accessible tributaries. The so-called Q-LV-Time is the interval from the onset of the QRS on the surface ECG to the sensed signal at the tip of the transvenous epicardial LV lead. This can be either expressed in milliseconds or as percentage of the QRS width on surface ECG. Zanon et al. evaluated whether Q-LV might be used to identify the optimal site in an individual patient by systematically screening all the suitable coronary sinus (CS) veins [40]. A strong correlation was observed between Q-LV prolongation and improvements in acute hemodynamic response. A Q-LV value of greater than 95 ms appeared significant, yielding an improvement in AHR of > 10%, a finding which has been associated with predicting long-term remodeling (Fig. 5). Certainly, hemodynamic effect could have been higher if the AV-delay were optimized in every site instead of using a fixed AV-delay of 130 ms.

Whether the study of BEHAR, performed in an exceedingly small group of patients (*N* = 8), all ischemic with a complex protocol and showing that the region of latest endocardial electrical activation does not reflect the best hemodynamic response, should change our implantation strategy is unclear [41].

Generally, when the latest electrical activation is detected, this spot should be the preferred implantation site except it is in a transmural scar.

In a previous study by Gold et al., the implantation of the LV lead with a favorable Q-LV interval was independently associated with a greater reverse remodeling and symptomatic improvement at 6 months [37]. In a double-blind randomized trial, it was recently shown that an electrically guided CRT implantation appeared non-inferior to an imaging-guided strategy as described before considering the outcomes of change in LVEF, LV reverse remodeling, and clinical response [42].

A general guide for successful CRT implantation can be found in Table 1.

**Table 1:** Criteria for optimal CRT response

Pre-implant	During implant	Post-implant
Patient selection	Identifying suitable tributaries	Save a 12-lead ECG of the intrinsic QRS-morphology
Optimal non-device HF treatment	Accept sometimes time-consuming repositioning for optimal target position	Vector optimization (QRS width, echocardiographic AV/VV optimization), avoid PNS
Identifying challenging implant cases	Know your tools and do not hesitate to use them	Echocardiographic AV/VV optimization if non-response with empirical programmed AV time
Characterizing the LV through non-invasive measures	Aim at the previously identified target area in complex cases	Eliminate reasons for insufficient BiV pacing (optimal rhythm control in AF, ablate PVC)
Identifying the intended optimal implantation site, multimodality imaging (cMRI, CT, EAM, ECGi)	In routine cases, stick to the empirical lateral/inferolateral position	Use adaptive algorithms
	Try to avoid apical/anterior lead placement	Regular device interrogations including 12-lead ECG, Holter ECG, exercise ECG, telemonitoring
	Add hemodynamic optimization if in doubt	Consider alternative approaches in case of non-response (HBP, endocardial LV pacing, surgical epicardial placement)
	Aim at long Q-LV interval	
	Use quadripolar leads/multipoint pacing	
	Avoid LV lead placement in scar tissue	

### Additional Considerations—with Respect to LV Pacing Site

#### LV only versus BIV

Early invasive studies intended to identify the best acute hemodynamic effect for different pacing sites did not show an acute difference for both pacing modes when stimulating the same site. LV pacing alone resulted in a significant functional improvement [31, 43].

Further trials like GREATER-EARTH and B-LEFT-HF showed neither inferiority nor superiority for LV over BIV during 6–12 months follow-up regarding echocardiographic reverse remodeling parameters, clinical and functional outcome parameters as well as biomarkers [44–46].

In a subgroup of CRT candidates with a normal AV conduction, an adaptive algorithm might provide intrinsic RV activation which results in a LV stimulation alone. Echocardiographic measurements showed an improved and potentially more efficient LV activation sequence without reducing dyssynchrony. Whether this has a detectable clinical benefit remains unproven [47•].

In summary, there are no acute, chronic, or clinical data which suggest that a different LV pacing site is favorable depending on an intended LV or BIV pacing mode. Thus, this is not to be considered during the implantation.

#### Multipoint Pacing (MPP)

Multipoint pacing (MPP) refers to pacing from more than one pole of a multipolar LV lead. Physiologically, this also results in “multifocal” or “trifocal” stimulation, albeit from only two leads.

Quadripolar CS leads offer the opportunity to program different vectors and activate larger areas of myocardium by cathodal stimulation from two widely separated electrodes. Besides the greater opportunities regarding threshold, intrinsic sensing and phrenic nerve stimulation multipoint pacing might be of superior benefit in extremely enlarged left ventricles and in non-responders as it might be suspected by retrospective analysis [48]. In acute hemodynamic measurements in typical LBBB patients, an adjunct benefit over conventional BIV pacing was proven at any site, but after optimization of BIV, MPP was not always superior. Thus, primarily the optimization of the LV pacing site is of greater relevance than MPP itself [49, 50•, 51].

Finally, a meta-analysis comparing MPP with BIP group suggested that MPP seems to be superior and decreased heart failure hospitalizations, improved LVEF, increased CRT response, and decreased all-cause morbidity and cardiovascular death rate [52].

#### Conduction System Pacing, HIS, and LBB Pacing

Biventricular pacing using the epicardial venous system is the most established approach to resynchronize the atrioventricular filling and the interaction between both ventricles. This approach has been studied extensively over more than two decades and the results in heart failure patients with a QRS > 130 ms and typical LBBB configuration are convincing and have been implemented in all guidelines. Nevertheless, biventricular pacing in other conduction disorders such as prolonged PR interval, QRS < 130 ms, and atypical LBBB or RBBB has shown a diverging or even deleterious effect. Right ventricular pacing itself has also a negative impact on cardiac function and survival in selected patients. Thus, the idea came up to access the original conduction system at different levels to allow a physiological electrical propagation.

Already in 1977, Narula et al. described a narrowing of QRS in a LBBB patient when the HIS bundle was stimulated [53]. Upadyay et al. found, using intracardiac septal mapping, that conduction block within the left‐sided His fibers or proximal portion of the left bundle branch was present in 64% of patients with a 12‐lead ECG appearance of LBBB [54]. These patients have the highest chance of successful resynchronization with His bundle pacing (HBP). Surface 12-lead ECG is not predictive to localize the region of propagation disturbance which makes patient selection critical without invasive evaluation [54, 55].

A short left ventricular activation (LVAT95) time as invasively investigated by Arnold et al. identifies patients in whom HBP does not result in a normalization of electrical propagation. Before suggesting HBP as a serious alternative to epicardial transvenous BIVP, it is important to distinguish disease conceptually and anatomically within the His–Purkinje conduction system from the appearance of propagation discontinuity on the LV epicardial surface [56].

Although the technique of HBP can be readily learned with a high level of success after an appropriate learning curve, its success is still adversely impacted by the atrioventricular (AV) nodal and infra-Hisian conduction disease [57–59]. Still, HBP is limited by poor ventricular sensing, elevated acute and chronic pacing thresholds necessitating lead reintervention and rapid battery replacement, and failure to reliably normalize the QRS complex in patients with bundle branch block. The benefits and limitations of HBP were evident in the pilot His-SYNC trial (His Bundle Pacing Versus Coronary Sinus Pacing for Cardiac Resynchronization Therapy) [7], which demonstrated in an on-treatment analysis that HBP resulted in superior electrical resynchronization with a trend toward improved echocardiographic response as compared with BIV; however, nearly one half of the patients randomized to the HBP arm required crossover to BIV, most commonly due to a failure to correct the wide QRS duration (> 150 ms) [60]. Targeting the region below HIS bundle is emerging as left bundle branch pacing (LBP). The idea behind this is to screw a lead through the interventricular septum until the left bundle can be stimulated, but without perforation of the septum. This method seems technically feasible with a success above 90%, roughly 10% injury of the right bundle branch and increase of tricuspid regurgitation initially described by Huang et al. and expanded by an observational prospective experience of more than 600 patients with a pacemaker indication [61, 62].

Summarizing, technically it is feasible to stimulate either the HIS bundle or the left bundle branch with improved tools, but it is too early to dethrone conventional transvenous biventricular or left ventricular pacing. The current evidence for HBP is superior to LBP, but both are still in their infancy compared to the long robust scientific evaluation of conventional BVP. Furthermore, it is still unclear which conduction disorder is treated best by which stimulation approach.

#### Epicardial versus Endocardial (WISE) Pacing—Retrograde versus Transseptal

Based on the physiological electrical propagation from the endocardial to the epicardial space, a primarily endocardial stimulation will be superior and might not be limited by the coronary venous anatomy. Whereas modern leadless pacing is mainly used in single chamber pacing of the right ventricle, recently allowing an AV-synchronous pacing triggered by heart sounds and not by the electrical atrial signal, left ventricular endocardial pacing is more challenging. In this context, the prospective, multi-center ALSYNC study proved feasibility of a transseptal approach to implant an endocardial LV lead with a considerable clinical outcome after 6 months. There are a few limitations, though. Transseptal puncture via the SVC may be challenging; a permanent lead in the LV is associated with a significant risk of thromboembolic events requiring long-term anticoagulation and mitral valve damage due to mechanical irritation or endocarditis. Therefore, a transvenous permanent endocardial approach should be limited to exceptional cases. To overcome the risk of thromboembolic events and to avoid permanent anticoagulation, a tiny receiver electrode of less than 10 mm length and 2.7 mm diameter was developed for the WISE-CRT system (EBR Systems, Sunnyvale, California). The receiver transforms ultrasound energy delivered from the transmitter which is implanted at the intercostal space and is connected to the battery. The system routinely uses an arterial femoral access with retrograde crossing of the aortic valve and needs a mandatory RV pacing electrode which is required for triggering. Further limitations are the distance between transmitter and receiver, the angulation between both, and an early battery depletion. Predominant complications are related to the femoral access and its closure. Based on the closure experience after TAVR procedures, this problem can be overcome in an interventional team, but a transseptal access has also been described if an arterial access is impossible [63].

The SELECT-LV trails as well as the real-world post-market registry have proven the technical feasibility but have also shown the potential risks and complications as perforation of the LV, induction of arrhythmias, and peripheral embolization of the receiver in one patient [64–66]. The clinical and reverse remodeling response was comparable to the standard biventricular pacing with approximately 70%. However, it has to be kept in mind that patients selected for the leadless ultrasound stimulation have been either clinical non-responder to standard BIV pacing or had no accessible cardiac veins.

Generally, this innovative technology offers promising LV stimulation options, but requires further improvement as the lack of RV pacing, simplification and miniaturization of delivery tools, and improvement of battery longevity.

#### How To Place a Left Ventricular Lead for Cardiac Resynchronization Therapy

Corrective pacing to improve the activation of electrically delayed left ventricular wall segments with subsequent dyssynchronous mechanical deformation is the cornerstone of cardiac resynchronization. In most patients with LBBB, the electro-mechanical delay is found most pronounced at the lateral left ventricular wall. Placement of left ventricular pacing leads for CRT is therefore mainly aimed at attaining a stable and electrically appropriate left ventricular lateral position of the pacing electrode(s). At the same time, intolerable unintended phrenic nerve stimulation must absolutely be avoided.

Several routes are possible to reach these goals. Since more than two decades, the standard way to obtain a left ventricular lateral electrode position is transvenous lead placement. The LV lateral wall is reached using lateral coronary venous branches draining to the main coronary sinus or the great cardiac vein [67].

**Fig. 6:**
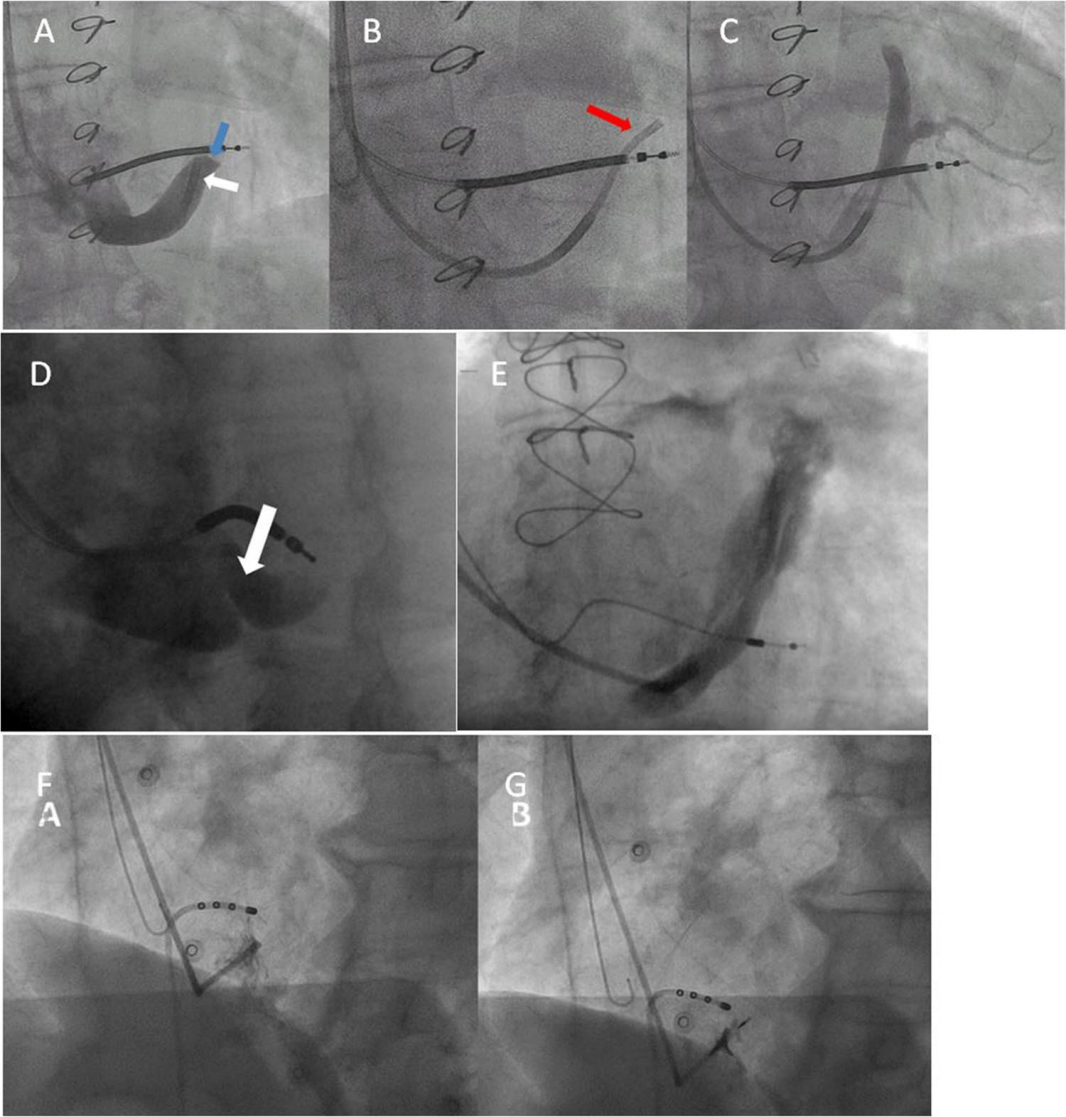
Fluoroscopic implantation images (**A**–**G**). **A**–**C** Venous valve at the mid-coronary sinus region (“Vieussens valve”), completely obstructing retrograde contrast flow (**A**, blue arrow indicates the valve; white arrow, guiding catheter tip). Introduction of a thin sub-selective catheter (red arrow, wire already retracted, also note the retraction of the guiding catheter), enabling cannulation beyond the valve (**B**) and definition of a suitable side branch anatomy for LV lead placement (**C**). **D** Ostial (“Thebesian”) coronary venous valve (white arrow). **E** Extended coronary sinus dissection after attempting to overcome a Vieussens valve obstruction with the catheter tip placed distally and ungentle wire advancement. **F**, **G** Left bundle branch lead placement in LAO view. The left panel (**F**) shows a small contrast injection defining the septal endocardium at the tip of the 3D implantation catheter distal and caudal to the His region (indicated by the quadripolar EP catheter). The right panel (**G**) shows deep septal implantation of a Medtronic 3830 SelectSecure™ lumenless pacing lead

Before the introduction of transvenous lead placement, open surgery and left ventricular epicardial lead implantation has been the standard way to pace the left ventricle [68]. This method is still in use, although no longer the standard technique. Epicardial leads are sutured to the epicardial surface or screwed into the myocardium. Alternative approaches to LV pacing that are still awaiting thorough evaluation comprise ultrasound-activated LV endocardial electrode and transseptal left bundle branch pacing (Fig. 6f, g) [69, 70].

#### Transvenous LV Lead Placement over Coronary Sinus Branches

Transvenous LV lead placement may be complex and requires specific training and experience. This has been addressed by structured education programs [71].

After introducing a 0.035 wire and a pre-shaped guiding sheath into the right heart, engagement of the coronary sinus ostium with an atraumatic wire followed by the guiding catheter is the first step to successful lead implantation. The CS ostium can be located by pulling back the catheter from the RV across the tricuspid valve while applying some counterclockwise torque. Immediately after crossing the tricuspid valve, the catheter moves posteriorly, and the tip reaches the CS ostial region. In many patients, the entry into the CS ostium is complicated by the presence of an ostial (“Thebesian”) valve (Fig. 6d), which enables blood drainage from the CS to the right atrium but impedes backflow to the CS and hinders the entry of the (frequently upward oriented) catheter tip. Usually, the guiding catheters have a multipurpose-like distal shape, but different catheter curves are available to fit individual anatomical properties and should be tried after unsuccessful attempts to cannulate the ostium. In many cases, a diagnostic left coronary Amplatz 1 or Amplatz 2 catheter (inserted over the 0.035-in. wire) helps to orientate the tip downward and allows getting beyond the Thebesian valve with the wire followed by the catheter. A second venous valve (Vieussens valve, Fig. 6a) is encountered at the mid-coronary sinus region in the majority of patients and may be a relevant obstacle to transvenous LV lead placement in up to 20% of patients [72]. The guiding catheter should only be passed through these valves on a guidewire, as blunt retrograde manipulation to overcome an obstructing Vieussens valve will likely result in extensive CS dissection (Fig. 6e). Sometimes the successful passage of a rigid valve may afford considerable effort and sophisticated experienced telescoping technique (Fig. 6b, c) resembling the treatment of a coronary artery chronic total occlusion. LV transvenous leads are usually implanted over a 0.014-in. guidewire. The angle of LV lateral veins with reference to their orifice to the CS is frequently sharp and may make it hard to advance a guidewire into the side branch and to overcome the kinked implantation route with the LV lead over the wire. Possible strategies to mitigate this problem include the use of firm guidewires and the placement of a second (“buddy”) wire. The most promising approach to lead implantation over sharp angles is the use of a sub-selective inner catheter with an angled tip. These catheters are advanced distally to the side branch orifice over a guide wire with or without a dilator. After drawback of the wire (and the dilator, if used), the sub-selective catheter enables side branch engagement through gentle pull-back and rotation. The threshold to use sub-selective inner catheters to cannulate angulated side branches should be low, as these catheters not only enable wire placement in difficult anatomies, but also provide the required back-up to advance the lead over the wire to the distal CS side branch.

The LV lead should be selected according to the side branch anatomy. Various hook-like or spiral shapes are available in different lengths (of the entire lead and the distal straight part). Stabilization within the vein is accomplished by advancing the tip into a distal wedge position. Guidewire withdrawal within the lead to the main CS releases the pre-shaped lead configuration and increases the lead adherence to the venous wall. Quadripolar leads should be preferred over bipolar or unipolar leads in order to increase the variability of pacing configurations, to avoid phrenic nerve stimulation, and to enable simultaneous multi-point pacing (MPP, see above) of the LV lateral myocardium.

In large straight vein branches or in redo procedures after lead dislodgement, with anticipated poor lead stability, a special active fixation lead (e.g., Medtronic Attain Stability Quad) is recommended. This lead is equipped with a sharp hook that is attached to the lead body at some distance proximal to the lead tip. By clockwise rotation, the lateral hook engages the venous wall firmly and stabilizes the electrode in a desired position.

An interesting additional strategy is the use of collateral veins to reach a desired pacing region. In many patients, the size of venous collaterals allows implanting conventional 4F leads over this way. A new promising technology, called LV micro-lead implantation, has been reported to enable implant quadripolar very thin leads over very thin collaterals using a dedicated wire core with four electrodes (1,2F) and a specific thin catheter (tip 2,4F) [73•].

**Fig. 7:**
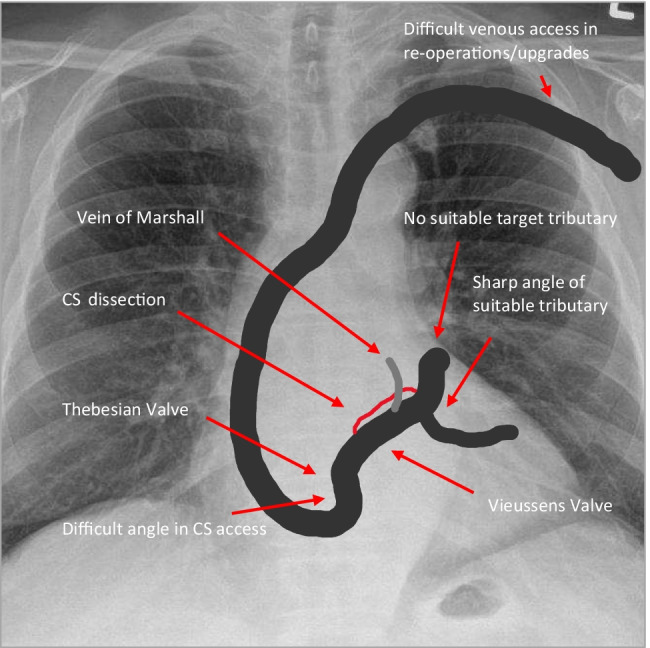
Schematic representation of pitfalls during LV lead implantation. Carelessly advancing sheaths over the Vieussens valve or accidental balloon occlusion in the Vein of Marshall may cause CS dissection

Interventional techniques like balloon dilatation and snaring of a guide wire may also help to overcome difficult coronary venous anatomies [74]. A schematic representation of pitfalls of LV lead implantation is shown in Fig. 7.

## Conclusion

**Practical recommendations—workflow:**

- **Transvenous epicardial approach:**

*Non-ischemic DCM with LBBB* > *150 ms without previous interventions*

LV implantations site less critical, but

- Based on venogram: avoid apical position, avoid anterior position.
- Target postero-lateral or lateral position (confirm in 2 angulations in fluoro).

*Ischemic cardiomyopathy with QRS 130–150 ms and not typical LBBB, re-implantations, chronic “non-responder”*

LV lead implant site crucial for response

- If implantation is only anatomical guided, target the same regions as in DCM, but
- Expend pre-interventional imaging (MRI, tissue Doppler imaging, CT) to detect scar and late mechanical activation.
- Avoid placing in a scar region.
- Identify target region by delayed electrical activation and verify with CS venogram the availability of tributary in aimed site.
- If more than one tributary will touch the target region, verify the superiority by acute hemodynamic response or shortening of the electrical delay using at least different AV-intervals.
- Narrowing of the paced surface QRS will additionally be helpful.

Generally, an individualized approach based on the acute improvement of hemodynamics and/or electrical parameters would be the goal to achieve the most benefit in each patient, but in routine these options are barely used.

### Alternate pacing options

If a target vein is not accessible, an alternate stimulation approach via the conduction system as HIS bundle or left bundle brunch pacing should be considered depending on the conduction disorder. A leadless endocardial left ventricular stimulation might be a further option.

## Abbreviations

BIV/BIVP: Biventricular pacing
CMRI: Cardiac magnetic resonance imaging
CRT: Cardiac resynchronization therapy
CS: Coronary sinus
CSP: Conduction system pacing
DSCT: Dual-source computed tomography
ECGI: Electrocardiographic imaging
HBP: His bundle pacing
LBBB: Left bundle branch block
LBP: Left bundle pacing
LEA: Latest electrical activation
LGE: Late gadolinium enhancement
LV: Left ventricle
MPP: Multipoint pacing
NYHA: New York Heart Association
QOL: Quality of life
PP: Pulse pressure
SDI: Systolic dyssynchrony index
TDI: Tissue Doppler imaging

## Declarations

NA
